# Adiponectin Related Vascular and Cardiac Benefits in Obesity: Is There a Role for an Epigenetically Regulated Mechanism?

**DOI:** 10.3389/fcvm.2021.768026

**Published:** 2021-11-19

**Authors:** Rosaria Anna Fontanella, Lucia Scisciola, Maria Rosaria Rizzo, Surina Surina, Celestino Sardu, Raffaele Marfella, Giuseppe Paolisso, Michelangela Barbieri

**Affiliations:** ^1^Department of Advanced Medical and Surgical Sciences, University of Campania “Luigi Vanvitelli”, Naples, Italy; ^2^Mediterrannea Cardiocentro, Naples, Italy

**Keywords:** adiponectin, obesity, inflammation, epigenetic mechanisms, cardiovascular complications

## Abstract

In obesity, several epigenetic modifications, including histones remodeling, DNA methylation, and microRNAs, could accumulate and determine increased expression of inflammatory molecules, the adipokines, that in turn might induce or accelerate the onset and development of cardiovascular and metabolic disorders. In order to better clarify the potential epigenetic mechanisms underlying the modulation of the inflammatory response by adipokines, the DNA methylation profile in peripheral leukocytes of the promoter region of IL-6 and NF-kB genes and plasma miRNA-21 levels were evaluated in 356 healthy subjects, using quantitative pyrosequencing-based analysis, and correlated with plasma adiponectin levels, body fat content and the primary pro-inflammatory markers. In addition, correlation analysis of DNA methylation profiles and miRNA-21 plasma levels with intima-media thickness (IMT), a surrogate marker for early atherosclerosis, left ventricular mass (LVM), left ventricular ejection fraction (LVEF), and cardiac performance index (MPI) was also performed to evaluate any potential clinical implication in terms of cardiovascular outcome. Results achieved confirmed the role of epigenetics in the obesity-related cardiovascular complications and firstly supported the potential role of plasma miRNA-21 and IL-6 and NF-kB DNA methylation changes in nucleated blood cells as potential biomarkers for predicting cardiovascular risk in obesity. Furthermore, our results, showing a role of adiponectin in preventing epigenetic modification induced by increased adipose tissue content in obese subjects, provide new evidence of an additional mechanism underlying the anti-inflammatory properties and the cardiovascular benefits of adiponectin. The exact mechanisms underlying the obesity-related epigenetic modifications found in the blood cells and whether similar epigenetic changes reflect adipose and myocardial tissue modifications need to be further investigated in future experiments.

## Introduction

### Obesity, Adipokines, and Inflammatory Markers

Inflammation has been shown to play a pivotal role in the genesis and development of atherosclerosis (1). In particular, increased body weight, dyslipidemia, and impaired gluco-metabolic control contribute to impair endothelial function by stimulating different cellular and bloodstream factors evoking an inflammatory response. In contrast, an appropriate lifestyle encompassing daily exercise, bodyweight control, Mediterranean diet, and strict glucose control have been shown to counteract the genesis and the negative impact of pro-inflammatory factors on endothelial functioning. Among the different bioactive substances secreted from fat tissue, the adipokines seem to play a key role in regulating inflammatory and immune responses (2). Adipokines related balance of pro-and anti-inflammatory cytokines/chemokines is made up of leptin, tumor necrosis factor-α (TNF-α), and interleukin-6 (IL-6) (3) (as pro-inflammatory factors) and adiponectin, which on the other hand, attenuates excessive inflammatory responses in a variety of tissues since it interferes with the functions of macrophages, T lymphocytes, and NK cells (4). Several clinical studies highlighted the inverse relationship between plasma adiponectin levels and several inflammatory markers, including C reactive protein. Adiponectin treatment reduces TNF-α-stimulated expression of E-selectin, vascular cell adhesion molecule-1 (VCAM-1), intracellular adhesion molecule-1, and IL-8 in human aortic endothelial cells and monocyte attachment to TNF-α-stimulated endothelial cells (5, 6). Adiponectin also inhibits TNF-α-induced nuclear factor-kB (NF-kB) activation in endothelial cells (6), an effect mediated by its ability to promote signaling through a cyclic AMP (cAMP)/protein kinase A (PKA) (4, 7). These anti-inflammatory properties of adiponectin could explain its beneficial effects on the cardiovascular apparatus. Notwithstanding, the complete molecular pathways by which adiponectin exerts its anti-inflammatory role needs to be more deeply investigated.

### Obesity, Epigenetic Modifications, and Inflammatory Modulation

Interestingly, various studies have suggested that different epigenetic marks could accumulate in obesity, including histone remodeling, DNA methylation, and microRNAs that alter the expression of inflammatory molecules that in turn might induce or accelerate the onset and development of cardiovascular and metabolic disorders (8, 9). To this regards, epigenome-wide DNA methylation association studies (EWAS) have identified differentially methylated CpG (dmCpG) loci that are associated with both adiposity (10–12) and related measures of inflammation (13, 14). Furthermore, fat tissue-related specific alterations in the expression pattern of miRNAs related to inflammatory processes (15) have also been demonstrated (16, 17). miRNA-21 is one of the most abundant and highly conserved recognized miRNAs, that is highly expressed in monocytes/macrophages (18), is involved in the modulation of the NF-kB pathway, and its tissues and bloodstream levels are associated with a variety of inflammatory conditions. However, the roles of miRNAs in the anti-inflammatory responses of adiponectin and, in particular, the epigenetic mechanisms underlying the adiponectin modulation of cytokines expression need further investigation.

### Is There a Role for an Epigenetically Regulated Mechanism in the Adiponectin-Related Inflammatory Modulation?

To better clarify the potential epigenetic mechanisms underlying the adiponectin modulation of cytokines expression, the DNA methylation profile in peripheral leukocytes of the promoter region of IL-6 and NF-kB genes and plasma miRNA-21 levels were evaluated in 356 healthy subjects and correlated with plasma adiponectin levels, body fat content and the primary pro-inflammatory markers. In addition, a correlation analysis of DNA methylation profiles and miRNA-21 plasma levels with intima-media thickness (IMT), a surrogate marker for early atherosclerosis, left ventricular mass (LVM), left ventricular ejection fraction (LVEF), and cardiac performance index (MPI) was also performed to evaluate any potential clinical implication in terms of cardiovascular outcome.

## Materials and Methods

### Subjects' Enrolment

Three hundred and fifty-six patients were recruited at the Internal Medicine and Geriatric Division of the University of Campania Luigi Vanvitelli from September 2017 to July 2019. Patients with NYHA class III-IV, cortisone therapy, previous or ongoing anti-tumor therapy, severe respiratory failure, continuous anti-inflammatory therapy, kidney or liver failure, infectious pathologies, autoimmune disorders, mental disorders, terminal diseases (SPV <6 months), and dementia were excluded from the study. Diabetes was diagnosed according to the American Association of Clinical Endocrinologists and the American Diabetes Association criteria (19). Patients answered a specific questionnaire about medicines used before the beginning of the study, the dates of the beginning and the end of treatment, the route of administration, and the duration of use. The study was approved by the ethics committee of the University of Campania, L. Vanvitelli, and informed written consent was obtained for each patient.

### Anthropometric and Bioelectrical Impedance Analysis Measures

Weight and height were measured by standard technique. Body mass index (BMI) was calculated as weight in kilograms divided by square of height expressed in meters. Waist circumference was measured at the midpoint between the lower rib margin and the iliac crest (most often at the umbilical level) and hip circumference at the level of the greater trochanter. Both were measured to the nearest 0.5-cm with a plastic tape measure, and the waist to hip ratio (WHR) was calculated. A standard mercury sphygmomanometer recorded baseline blood pressure. The disappearance of sound (phase V) was used for diastolic reading. All determinations were performed three times, two min apart while the subject was supine, on three occasions separated by 2 min of intervals. The average of the last two recorded measurements was considered in the analysis. Bioelectrical impedance BIA 101 BIVA device (Akern/RJL, Florence, Italy), measuring the resistance and reactance, was used to determine body composition as to fat, fat-free, lean, and muscular masses and phase angle with the participant lying relaxed; arms and legs were not in contact with other body parts. The legs were 45° apart, and the arms were 30° from the torso. Sensing electrodes were placed over the right wrist and ankle; current electrodes were placed over the metacarpals and metatarsals.

### IMT of Carotid Artery

For the evaluation of IMT, carotid sonography was measured by using a single ultrasound machine (PHILIPS iE33, Eindhoven, The Netherlands). The same physician performed the examinations following the standard protocol. Common carotid arteries, bifurcation, and internal carotid arteries were measured by ultrasound in all patients. The measurements of IMT from the right and left sides were made on the far wall, which was defined as the vertical distance from the leading edge of the first echogenic line to the second echogenic line. Three determinations of IMT were conducted, i.e., at the site of most significant thickness and the two other points, 1 cm upstream and 1 cm downstream from the side of the most significant thickness. The measurement values were averaged, and the averaged IMT value was adopted as the representative data for each measurement. The intra-individual and inter-individual CV of technique were 2.4 and 3.3%, respectively.

### Echocardiography

Transthoracic full two-dimensional and Doppler echocardiography assessment was performed at baseline and after 12 months, according to the American Society of Echocardiography recommendations (17). For echocardiography, we used a Philips iE33 echocardiography (Eindhoven, The Netherlands). LVM was calculated and normalized by both body surface area (BSA) and by height squared correctly for the effect of overweight (20). LVEF was calculated, dividing the stroke volume by the volume of blood collected in the left ventricle at the end of diastolic filling as end-diastolic volume (17). The stroke volume was the fraction of chamber volume ejected in systole as the difference between end-diastolic volume and end-systolic volume (17). Subsequently, we measured Doppler velocities and time intervals from mitral inflow and left ventricular outflow recordings. Mitral early diastolic flow deceleration time was calculated as the time interval between the peak of early diastolic velocity and the end of the early diastolic flow (17). The total systolic time interval was measured from the cessation of one mitral flow to the beginning of the following mitral inflow. The ratio of velocity-time intervals of mitral early and late diastolic flows was then calculated (17). The following myocardial Doppler indexes and intervals were also evaluated: the left ventricle isovolumetric relaxation time (IRT), left ventricle ejection time (ET) and left ventricle isovolumetric contraction time (ICT). The IRT was the time interval from the cessation of left ventricular outflow to the onset of mitral inflow. The ET was the left ventricle ejection time as an interval from the onset and cessation of left ventricular outflow (21). The ICT was calculated by subtracting ET and IRT from the total systolic time interval (21). Myocardial performance index (MPI) was calculated by using the formula MPI = (IRT + ICT)/ET (22). The MPI, by including both systolic and diastolic time intervals, could be evaluated for assessment of cardiac dysfunction (21). Myocardial Performance Index (MPI/Tei Index), which includes both systolic and diastolic time intervals to assess the global cardiac dysfunction, was used by Tei and his co-workers in 1995 (21). Tei Index uses the measurement possible on flow wave Doppler and is as sensitive as the tissue Doppler measurements. The index is used primarily in amyloidosis, dilated cardiomyopathy, ischemic heart disease, and congestive heart failure. Finally, we calculated in all study populations the LVM using two-dimensional (2D) echocardiography and via M-mode as recommended method (17). The used formula to estimate LVM from linear dimensions, based on the assumption of the left ventricle as a prolate ellipsoid of revolution, was to have linear measurements of inter-ventricular septum wall thickness (IVST), as well as left ventricular internal diameter (LVID) and posterior wall thickness (PWT), from the parasternal acoustic window in end-diastole at the level of the LV minor axis (mitral valve leaflet tips) using 2D-targeted M-mode or directly from 2D images (17).

### Laboratory Methods

After an overnight fast, plasma glucose, HbA1c, serum lipids were measured by enzymatic assays. Serum glucose was determined by an enzymatic colorimetric assay using a modified glucose oxidase-peroxidase method (Roche Diagnostics, GmbH, Mannheim, Germany) and a Roche-Hitachi 917 analyzer. Commercial enzymatic tests were used to determine serum total- and HDL- cholesterol and triglyceride levels (Roche Diagnostics). Serum LDL cholesterol levels were calculated by the Friedewald formula (23). The intraassay coefficient of variation was <3.8% for total cholesterol, less 5.0% for HDL cholesterol, <2.5% for triglycerides.

### Adiponectin and IL-6 and TNF-α ELISA Assay

The whole blood was centrifugated (1,000 g at 4°C for 10 min) to obtain plasma. Adiponectin, IL-6, and TNF-α assays were performed using the HUMAN ADP/Acpr30 ELISA Kit Elabscience (Cat #E-EL-H6122), Human IL-6 ELISA KIT Diaclone (Cat #950.030.192), and TNF-α ELISA KIT DIACLONE (Cat #950.090.192) according to manufacturer's protocols respectively.

### Isolation of PWBCs

Blood samples were collected in BD vacutainer EDTA tube. PWBCs were isolated by density-gradient centrifugation on Ficoll-Paque PLUS (GE Healthcare, Chicago, Illinois, United States). After centrifugation (400 x g for 20 min at 4°C), the interface cells were carefully removed and transferred to a new conical tube. PWBCs were washed twice in PBS, centrifuged at 300 g for 10 min at 4°C.

### Methylation Analysis

According to the manufacturer's protocols, DNA was extracted using the QIAamp DNA Blood Mini Kit (Qiagen, cat no. 51104). The methylation analysis of genes was investigated by pyrosequencing-based methylation analysis, using the PyroMark Q48 Autoprep (Qiagen) after DNA bisulfite conversion. Bisulfite conversion was performed with 350 μg of DNA isolated using the EZ DNA Methylation kit (Zymo Research, Irvine, California, United States, cat no. D5001) as recommended by the manufacturer. The bisulfite modified DNA was amplified by polymerase chain reaction (PCR), using the PyroMark PCR Kit (Qiagen, cat no. 978703). According to the manufacturer's instructions, each reaction mixture contained two μL of bisulfite-converted DNA, 12.5 μL of PyroMark PCR Master Mix 2X, containing HotStartTaq DNA Polymerase, 2.5 μL of Coral Load Concentrate 10X, and 2.5 μL of mix PCR primers (one of them was biotinylated). Optimized PCR cycling conditions were one cycle at 95°C for 15 min; 40 cycles at 94°C for 30 s, 56°C for 30 s, and 72°C for 30 s; and a final extension at 72°C for 10 min. Electrophoresis of the PCR products was performed on a 2% Agarose Gel (Amersham Biosciences). The biotinylated PCR products were subjected to sequencing using a PyroMark Q48 Advanced CpG Reagent (Qiagen, cat no. 974022) and analyzed by PyroMark CpG SW 1.0 software (Qiagen). All samples were run in triplicate to rule out experimental bias or some random error. The primers were commercially designed, and codes are listed below: *NF-*κ*B*: *Island n*°*1 in gene promoter*: Hs_ NF-kB1_01_PM PyroMark CpG assay (PM00110908)—bp 103423134—103423182 CRCh37/hg19; for IL-6 methylation study were used PCR and sequencing custom primers: PCR forward primer (5-AGGGATAATTTAGTTTAGAGTTTATTTGT-3), PCR reverse primer (biotin-5-CTCCCTCTCCCTATAAATCTTAATT-3) and sequencing primer (5-ATAAGAAATTTTTGGGTGT-3).

### miRNA Detection

According to the manufacturer's manufacturer, total RNA, including small RNAs, was isolated and purified from the buffy coat of patients using miReasy Mini Kit (Cat #217004; Qiagen, Hilden, Germany) instruction for cells samples. The RNA concentration and purity were detected using a QIAexpert spectrophotometer (Cat #1038703; Qiagen). Then complementary DNA (cDNA) was synthesized from 10 ng of total RNA using a TaqMan MicroRNA Reverse Transcription Kit (Cat #4366597; Applied Biosystem Lithuania) with a specific RT primer (Cat #4440887 hsa-miR21-5p; Cat #4427975 U6 snRNA; Applied Biosystem, Lithuania) according to the manufacturer's protocol. miRNA expression was measured with a Rotor-Gene Q (Cat #R0515102; Qiagen) using PrimeTime Gene Expression Master Mix (Cat #1055772; IDT) and the TM primer (Cat #4440887 hsa-miR21-5p; Cat #4427975 U6 snRNA Applied Biosystem). The two-step PCR condition was 3 m at 95°C, 45 cycles with 5 s at 95°C, and 30 s at 60°C (10 μl reaction volume and 2 μl cDNA template). All samples were run in triplicate. A threshold cycle (*C*_t_) value was obtained for each amplification cycle, and Δ*C*_t_ was calculated as the *C*_t_ difference between target miRNA and U6. miRNA expression was calculated using the 2^−ΔΔ*Ct*^ method.

### Statistical Analysis

For investigating the difference between groups, the sample size was estimated on an IBM computer by GPOWER software. The resulting sample size, calculated according to a global effect size of 27% with a type I error of 0.05 and a power of 95%, was 210 patients. Differences between groups were compared using a one-way analysis of variance (ANOVA) test. Differences between groups were considered significant at a *P*-value of < 0.05. Correlation analyses were performed using Pearson or Spearman correlation coefficients, as appropriate. The independent association of adiponectin with DNA methylation levels was tested in multivariate analysis. Statistical analyses were performed using SPSS v23 software (IBM SPSS®, Chicago).

## Results

Clinical characteristics of patients enrolled are presented in Table 1. Categorizing subjects in obese and non-obese subjects, obese subjects were older, had a higher percent body fat content, higher arterial blood pressure, total cholesterol level, and lower adiponectin plasma levels. Furthermore, obese subjects had more increased IMT, LVM, LVM Index and MPI compared to non-obese subjects. No difference in LVEF was found between obese and non-obese subjects. Concerning plasma markers of systemic inflammation, obese subjects had significantly higher plasma fibrinogen, PCR, and IL-6 levels (Table 1). In all study population, plasma adiponectin levels significantly correlated with body fat content (*r* = −0.250; *p* < 0.05), WHR (*r* = −0.129; *p* < 0.05) PCR (*r* = −0.137; *p* = 0.03), TNF-α levels (*r* = −0.209; *p* = 0.01), IMT (−0.21; *p* < 0.005), LVM (−0.180; *p* < 0.04) and MPI (−0.238; *p* < 0.003).

**Table 1:** Clinical characteristic of study population (*n* = 356).

	**Non-obese (*n* = 233)**	**Obese (*n* = 123)**
Age (years)	64.11 ± 1.18	68.12 ± 0.86^*^
Sex (M/F)	127/106	67/56
BMI (kg/m^2^)	25.67 ± 0.19	34.96 ± 0.48^*^
Fat Mass (%)	30.51 ± 1.98	42.65 ± 1.24^*^
Systolic arterial pressure (mmHg)	126.33 ± 0.93	132.85 ± 1.42^*^
Dyastolic arterial pressure (mmHg)	77.83 ± 0.59	83.36 ± 1.19^*^
Triglycerides (mmol/L)	123.03 ± 6.94	134.35 ± 6.23
Cholesterol (mmol/L)	184.41 ± 3.09	187.88 ± 3.93^*^
HDL (mmol/L)	52.76 ± 1.12	48.49 ± 1.34
LDL (mmol/L)	107.61 ± 2.74	112.87 ± 3.52
Plasma glucose level (mg/dL)	106.94 ± 3.03	111.29 ± 4.46
Diabetes (%)	32.6	34.1
Hypertension (%)	69.5	76.4
Dyslipidemia (%)	33.5	44.7^*^
Steatosis (%)	28.3	56.1^*^
Ischemic heart disease (%)	13.7	13.0
IMT	0.79 ± 0.01	0.92 ± 0.08^*^
LVM (g)	138.8 ± 0.63	178.5 ± 0.55^*^
LVmass/BSA (g/m^2^)	59.8 ± 16.2	78.3 ± 17.5^*^
LVEF	55.3 ± 0.49	58.2 ± 0.77
MPI	0.35 ± 0.01	0.56 ± 0.01^*^
Interleukin-6 (pg/mL)	15.94 ± 2.49	25.91 ± 3.76^*^
TNF-a (pg/mL)	15.5 ± 1.5	17.0 ± 2.26
Adiponectin (pg/mL)	12.71 ± 0.67	9.51 ± 0.90^*^
C-reactive Protein (mg/dL)	0.7 ± 0.15	1.26 ± 0.19^*^
Fibrinogen (mg/dL)	332.73 ± 6.73	357.58 ± 11.02^*^

*Values are mean ± SE*.

* *p < 0.05 vs. non-obese subjects. IMT, intima media thickness; LVM, left ventricular mass; LVEF, left ventricular ejection fraction; MPI, myocardial performance index*.

### IL-6 DNA Methylation Analysis

**Figure 1:**
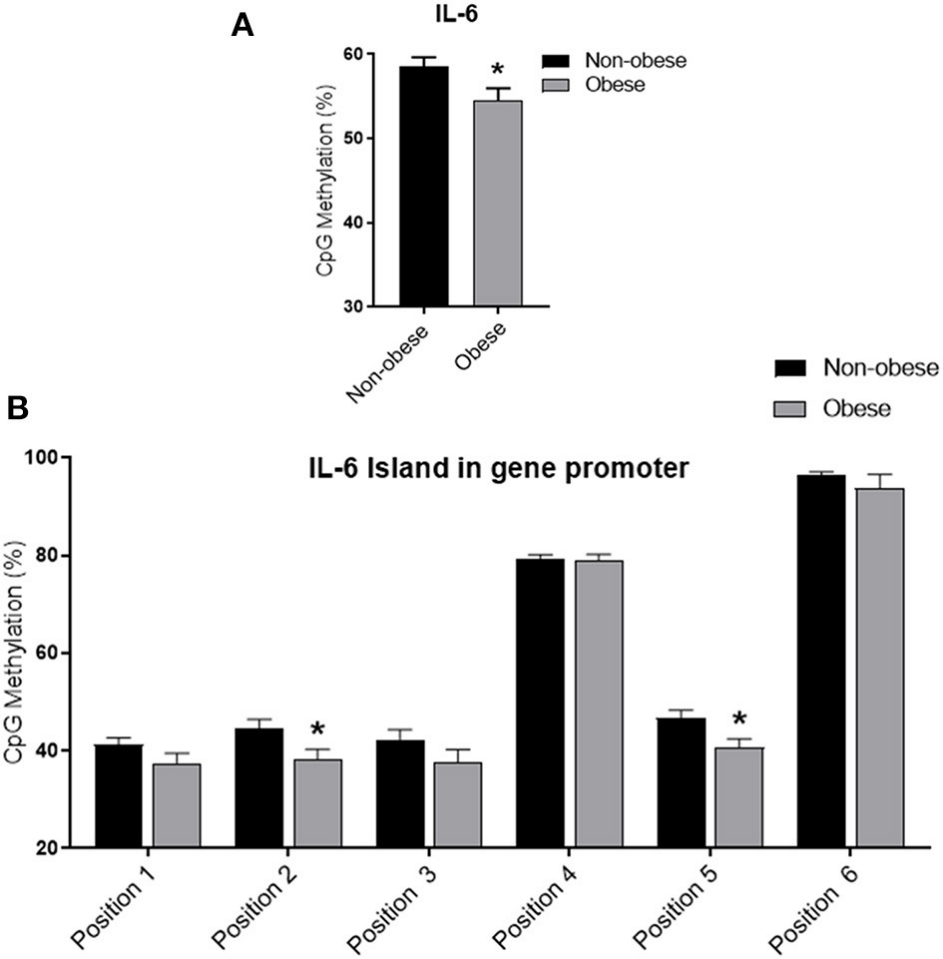
Methylation analysis of IL-6 promoter in obese and non-obese subjects **(A)** Schematic representation of CpG site locations in human IL-6 promoter. **(B)** The DNA methylation status was expressed as percentage of CpGs methylation. Data are mean ± Standard Errors. *p < 0.05 vs. non-obese subjects.

Obese subjects showed statistically significantly lower mean *IL6* DNA methylation levels of the promoter region compared to non-obese patients (*p* < 0.05). In particular individual analyses of the positions studied demonstrated that obese patients exhibit significantly lower methylation levels in positions 2 and 5 (*p* < 0.05) (Figure 1). In all study populations, mean *IL-6* DNA methylation levels were inversely correlated with body fat content (*r* = −0.314; *p* = 0.01) and positively correlated with adiponectin plasma levels (*r* = 0.202; *p* = 0.016). A negative correlation between *IL-6* DNA methylation levels, IL-6 plasma levels (*r* = −0.460; *p* < 0.001), IMT (*r* = −0.221; *p* < 0.005), and MPI (*r* = −0.346; *p* < 0.005), was also found. Correlation analyses in each position investigated between adiponectin, body fat content, and plasma IL-6, IMT, and echocardiographic parameters with IL-6 DNA methylation levels are shown in Supplementary Table 1. The potential predictive role of adiponectin and body fat content on *IL-6* DNA methylation level was tested in a multivariate analysis. In a model including age, sex, arterial blood pressure, dyslipidemia, diabetes, steatosis, and drug use, only sex, adiponectin plasma levels, and obesity resulted significant predictors of IL-6 DNA methylation, independently of the other covariates (Table 2). A model encompassing also the interaction term “adiponectin^*^obesity” revealed the interaction term independently associated with IL-6 DNA methylation (Beta = −0.797; *t* = −2.134, *p* < 0.05).

**Table 2:** Linear regression analysis with IL-6 DNA methylation as the dependent variable.

	**IL-6 DNA methylation**		
**Variables**	**Beta**	***t***	***p***
Age (years)	−0.068	−0.508	0.614
Sex (M/F)	**0.373**	**3.160**	**0.003**
Dyslipidemia	−0.070	−0.543	0.590
Diabetes	−0.142	−1.137	0.262
Obesity	**−0.312**	**−2.243**	**0.030**
Blood pressure	0.141	1.139	0.261
Fat mass	−0.003	−0.022	0.982
Adiponectin	**0.428**	**3.393**	**0.002**
Steatosis	0.073	0.564	0.576
Drug use^*^	−0.177	−1.332	0.190

* *Calculated as “users vs. non-users of at least one or more of the following medications: ACE inhibitors/sartans, diuretics, β-blockers, lipid-lowering drugs, hypoglycaemic drugs”. The bold values are statistically significant*.

### NF-kB DNA Methylation Analysis

**Figure 2:**
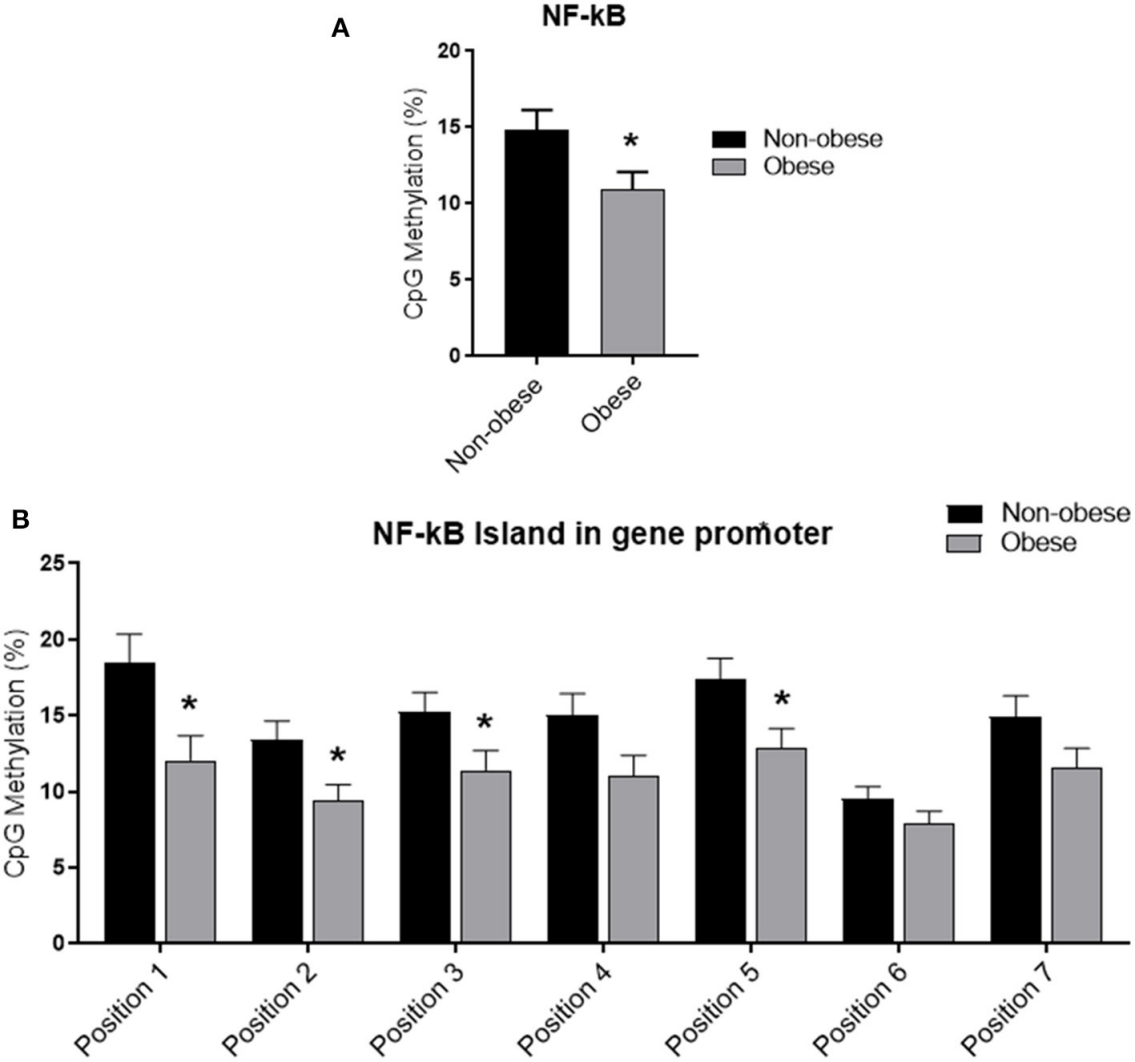
Methylation analysis of NF-kB promoter in obese and non-obese subjects **(A)** Schematic representation of CpG site locations in human NF-kB promoter. **(B)** The DNA methylation status was expressed as percentage of CpGs methylation. Data are mean ± Standard Errors. *p < 0.05 vs. non-obese subjects.

Categorizing subjects in obese and non-obese, obese subjects showed statistically significant lower levels of mean NF-kB DNA methylation of the promoter region compared to non-obese patients (*p* < 0.05). In particular, analyzing individually the positions studied, obese patients exhibit significantly lower methylation levels in four out of the seven positions investigated (Figure 2). In all study population, mean *NF-kB* DNA methylation levels were positively correlated with adiponectin plasma levels (*r* = 0.325; *p* < 0.003) and negatively correlated with plasma TNF-α levels (*r* = −0.273; *p* = 0.006). An inverse correlation between NF-kB DNA methylation levels, IMT (*r* = −0.238; *p* = 0.004), and MPI (*r* = −0.303; *p* = 0.003) was also found. Correlation analysis between adiponectin, TNF-α, IMT, and echocardiographic parameters with mean NF-kB DNA methylation and the methylation level of each position investigated are shown in Supplementary Table 2. The potential predictive role of adiponectin on NF-kB DNA methylation level was tested in a multivariate analysis. A model including age, sex, arterial blood pressure, obesity, diabetes, dyslipidemia, steatosis and drug use, only diabetes, plasma adiponectin levels, resulted in significant predictors of NF-kB DNA methylation independently of the other covariates (Table 3).

**Table 3:** Linear regression analysis with NF-kB-DNA Methylation as the dependent variable.

	**NF-kB DNA methylation**		
**Variables**	**Beta**	***t***	**Sign**
Age (years)	0.107	1.514	0.131
Sex	−0.045	−0.665	0.507
Dyslipidemia	−0.004	−0.055	0.956
Diabetes	**−0.148**	**−2.113**	**0.036**
Obesity	−0.125	−1.828	0.069
Blood pressure	−0.005	−0.066	0.948
Adiponectin	**0.158**	**2.303**	**0.022**
Steatosis	−0.032	−0.437	0.663
Drug use^*^	−0.043	−0.618	0.537

* *Calculated as “users vs. non-users of at least one or more of the following medications: ACE inhibitors/sartans, diuretics, β-blockers, lipid-lowering drugs, hypoglycaemic drugs”. The bold values are statistically significant*.

### miRNA 21

**Figure 3:**
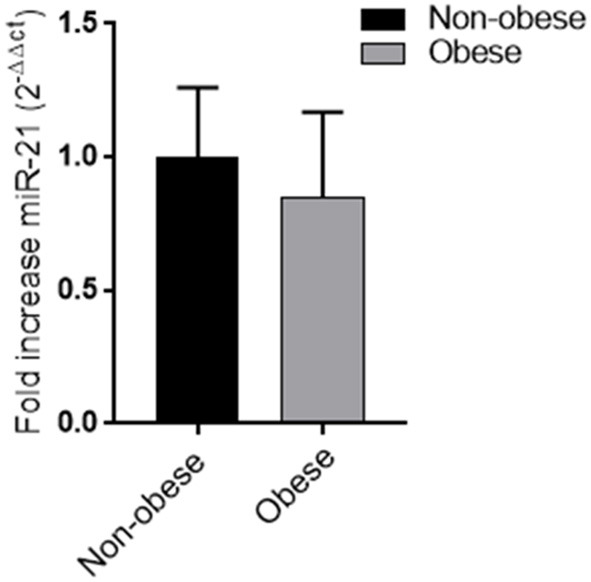
Plasma miRNA-21 levels in obese and non-obese subjects (*n* = 356) Data are mean ± Standard Errors.

No difference in miRNA 21 levels between obese and non-obese subjects was found (Figure 3). In all study population, plasma miRNA 21 levels negatively correlated with adiponectin plasma levels (*r* = −0.310; *p* = 0.002) and positively correlated with TNF-α levels (*r* = −0.214; *p* = 0.015) percent in body fat content (*r* = −0.308; *p* = 0.018), IMT (*r* = 0.292; *p* < 0.001) and MPI (*r* = 0.234; *p* < 0.05). A multivariate analysis with age, sex, arterial blood pressure, dyslipidemia, diabetes, steatosis, drug use, and obesity as covariates demonstrated that adiponectin and body fat content were independent predictors of plasma miRNA 21 (Table 4).

**Table 4:** Linear regression analysis with miRNA-21 as the dependent variable.

	**miRNA-21**		
**Variable**	**Beta**	***t***	**Sign**
Age (years)	0.101	0.723	0.476
Sex (M/F)	**−0.284**	**−2.026**	**0.052**
Dyslipidemia	−0.080	−0.483	0.633
Diabetes	−0.055	−0.359	0.723
Obesity	0.041	0.259	0.797
Blood pressure	−0.030	−0.198	0.845
Fat mass	**0.310**	**2.007**	**0.048**
Adiponectin	**−0.410**	**−2.633**	**0.014**
Steatosis	0.146	0.871	0.391
Drug use^*^	0.104	0.699	0.491

* *Calculated as “users vs. non users of at least one or more of the following medications: ACE inhibitors/sartans, diuretics, β-blockers, lipid-lowering drugs, hypoglycemic drugs”. The bold values are statistically significant*.

## Discussion

Our study firstly demonstrates that obese subjects have statistically significantly lower levels of mean DNA methylation of the IL6 and NF-kB promoter regions compared to non-obese patients. Both IL-6 and NF-kB DNA methylation positively correlate with plasma adiponectin levels. Although no difference in plasma miRNA-21 between obese and non-obese subjects was found, a significant association between plasma miRNA-21 levels with adiponectin plasma levels and percent of body fat content was found. Finally, DNA methylation of the IL-6 and NF-kB promoter regions and plasma miRNA21 significantly correlated with IMT, a surrogate marker for early atherosclerosis, and MPI index, an echocardiographic parameter of cardiac dysfunction.

These results confirm that obesity could accumulate epigenetic marks, including DNA methylation and microRNAs plasma changes that, in turn, might alter the expression of inflammatory molecules and suggest for the first time a role of specific epigenetic modifications as a potential mechanism underlying the anti-inflammatory properties and cardiovascular benefits of adiponectin.

Increasing evidence indicates that obesity is closely associated with epigenetic modifications and that disturbances in DNA methylation of genes involved in lipid and lipoprotein metabolism, substrate transport, and inflammatory pathways predict the development of obesity-related disorders like T2D and cardiovascular diseases (20, 24). Furthermore, it has been reported that the alterations of the adipocyte physiology in obesity might also be related to specific alterations in the expression pattern of miRNAs related to inflammatory processes (25). Interestingly, in our study, obese subjects showed a low IL-6 and NF-kB DNA methylation level of the promoter regions compared to non-obese subjects. In agreement, the lower promoter methylation profiles found in obese subjects were associated with higher pro-inflammatory cytokines IL-6 and TNF-α plasma levels that play crucial roles in an obesity-associated increased risk of diabetes and cardiovascular diseases (26). Consistent with our data, previous studies have demonstrated that promoter methylation is an essential epigenetic mechanism of plasma IL-6 regulation (27) and that the IL-6 promoter is hypomethylated in white blood cells (WBCs) of patients affected by chronic inflammatory diseases (22). Indeed, the low methylation level of the NF-kB gene promoter, which corresponds to a transcriptionally active NF-kB gene (28) might explain the association found with TNF-α plasma levels. The transcription factor NF-kB induces, in fact, the expression of pro-inflammatory genes, including TNF-α and others cytokines and chemokines, and also regulates multiple aspects of innate and adaptive immune functions (29). Furthermore, in healthy populations, inter-individual variations in IL-6 and NF-kB DNA methylation from WBCs have been associated with risk factors for cardiovascular and metabolic disorders (18, 29). More intriguingly, although there was no difference between obese and non-obese subjects, body fat content positively correlated with miRNA-21 levels. Furthermore, a significant positive association between miRNA-21 and plasma TNF-α levels was found. miRNA-21 is among the most abundant and highly conserved recognized miRNAs, which is highly expressed in monocytes/macrophages (18) is involved in the modulation of the NF-kB pathway, and its tissues and plasma levels are associated with a variety of inflammatory conditions. miRNA-21 orchestrates the fine-tuning of the inflammatory response through direct and indirect activities on NF-kB pathways in a context-dependent manner.

Data evaluating whether DNA methylation changes in PBCs or mi-RNA plasma levels can serve as applicable, informative biomarkers for different health outcomes is rapidly emerging (30). Previous reports have indicated that miRNA-21 is associated with metabolic syndrome and is implicated in human adipose tissue-derived mesenchymal stem cell (hASC) proliferation and differentiation (31). Interestingly enough, in our study, DNA methylation of the IL-6 and NF-kB promoter regions and plasma miRNA-21 significantly correlated with IMT, a surrogate marker for early atherosclerosis, and MPI index, an echocardiographic parameter of cardiac dysfunction. These results confirm the role of epigenetics in the obesity-related cardiovascular complications and firstly support the potential role of plasma miRNA-21 and IL-6 and NF-kB DNA methylation changes in nucleated blood cells as potential biomarkers for predicting cardiovascular risk in obesity. The exact mechanism underlying the epigenetic modifications found in the blood cells of obese people and whether similar obesity-related epigenetic changes in PBCs are present in adipose and myocardial tissue need to be further investigated in a future experiment.

Several studies have demonstrated that adiponectin is a modulator of multiple obesity-linked diseases by attenuating excessive inflammatory responses in a variety of tissues (5, 6). Indeed, researches over the years have also highlighted a paradoxical effect on inflammation (32), demonstrating high levels of adiponectin in the presence of an important inflammatory state (33). Interestingly, application of the synthetic adiponectin receptor agonist AdipoRon has been recently shown to attenuate impairment of myocardial function, in a rat model of CPB-induced SIRS, by modulating systemic and myocardial inflammation. Indeed, the involvement of epigenetics factors in adiponectin-mediated suppression of cellular inflammatory responses has not been established. Recent reports suggested that miRNAs regulated by adiponectin act as mediators for anti-inflammatory action in adipose tissue (34). However, the role of modulation of miRNA-21 in the anti-inflammatory responses induced by adiponectin or the effects of adiponectin on IL-6 and NF-kB gene methylation has not been previously demonstrated yet. In a large population (*n* = 356) and using quantitative pyrosequencing-based analysis, our results showed an independent predictive role of adiponectin on IL-6 and NF-kB DNA methylation levels firstly, thus supporting the hypothesis of an involvement of epigenetic factors in the anti-inflammatory effect of adiponectin. Pyrosequencing-based analysis was suitable for measuring subtle methylation changes at more than one CpG site and thus for detecting DNA methylation more accurately in the region (35–37). Unfortunately, our data non-allow us to determine which epigenetic mediators of DNA methylation modifications (DNMTs, TET, etc.) are involved, and further specifically designed experimental studies will be necessary. As far as the independent effect of sex on IL-6 DNA methylation levels, results found are consistent with previous studies showing a sex difference in IL-6 expression (38). Interestingly enough, in our study population, an independent predictive role of adiponectin on plasma miRNA-21 levels was also demonstrated. In particular, an inverse relationship between adiponectin and miRNA-21 plasma levels was found. Previous *in vitro* data showed that miRNA-21 expression is promoted by TNF-α, IL-6, leptin, resistin, and FFAs in human adipocytes (39) and globular adiponectin in RAW 264.7 macrophages (40). Furthermore, the overexpression of miRNA-21 can significantly promote adipocytes differentiation and increase the expression of adiponectin. Our data non-allow us to determine whether plasma miRNA-21 changes are a consequence or cause of adiposity, hypo-adiponectin, and related inflammation and seems to contradict previous “*in vitro*” results. Indeed, “*in vitro*” and “*in vivo*” studies on animal models also suggested that miRNA-21 may act as a switch from early pro-inflammatory to anti-inflammatory activities of macrophages (41), both promoting or inhibiting NF-kB/NLRP3 pathways (42) and demonstrated that miRNA-21 is both a target and a regulator of ERK/NF-kB pathway (43).

In conclusion, our results firstly demonstrate a potential role of adiponectin in preventing epigenetic modification induced by increased adipose tissue content in obese subjects and suggest an additional mechanism underlying the anti-inflammatory properties and the cardiovascular benefits of adiponectin. Further studies are necessary to better elucidate the exact mechanisms underlying the obesity-related epigenetic modifications found in the blood cells and whether similar epigenetic changes in PBCs reflect adipose and myocardial tissue modifications.

## Data Availability Statement

The raw data supporting the conclusions of this article will be made available by the authors, without undue reservation.

## Ethics Statement

The studies involving human participants were reviewed and approved by the Ethics Committee of Università degli Studi della Campania Luigi Vanvitelli—Azienda Ospedaliera Universitaria Luigi Vanvitelli—AORN Ospedali dei Colli. The patients/participants provided their written informed consent to participate in this study.

## Author Contributions

MB, RF, LS, GP, MR, and RM designed research, data acquisition and analysis, interpretation of data, and wrote the paper. LS, RF, and SS performed research and analyzed data. CS contributed to enrolment and clinical evaluation of patients. All authors significantly contributed to the manuscript and approved the final version for publication.

## Funding

This study was supported by the Ministero dell'Istruzione, dell'Università e della Ricerca Scientifica (grants PRIN 2017).

## Conflict of Interest

The authors declare that the research was conducted in the absence of any commercial or financial relationships that could be construed as a potential conflict of interest.

## Publisher's Note

All claims expressed in this article are solely those of the authors and do not necessarily represent those of their affiliated organizations, or those of the publisher, the editors and the reviewers. Any product that may be evaluated in this article, or claim that may be made by its manufacturer, is not guaranteed or endorsed by the publisher.
