# Supplementary material for: Adiponectin Related Vascular and Cardiac Benefits in Obesity: Is There a Role for an Epigenetically Regulated Mechanism?
Source: Front Cardiovasc Med. 2021 Nov 19;8:768026. doi: 10.3389/fcvm.2021.768026 (PMC8639875; doi:10.3389/fcvm.2021.768026)
Supplement: Supplementary file 1 [file Table_1.DOCX]

**Supplementary table 1. Simple correlation analyses between adiponectin, percent of body fat content, IL-6 plasma levels, IMT and MPI**

**and IL-6 DNA methylation (n=356)**

|  | | **Mean Met-IL-6** | **Met- pos.1** | **Met pos. 2** | **Met- pos. 3** | **Met- pos. 4** | **Met- pos. 5** | **Met- pos. 6** |
| --- | --- | --- | --- | --- | --- | --- | --- | --- |
|  | |  |  |  |  |  |  |  |
| **Adiponectin** | *r* | **.202** | **.237** | .151 | .159 | **.281** | .077 | .114 |
|  | *p* | .016 | .005 | .073 | .058 | .001 | .360 | .177 |
|  |  |  |  |  |  |  |  |  |
| **Fat mass** | *r* | **-.314** | **-.326** | -.246 | -.198 | **-.290** | **-.326** | -.077 |
|  | *p* | .015 | .011 | .058 | .130 | .025 | .011 | .556 |
|  |  |  |  |  |  |  |  |  |
| **IL -6** | *r* | **-.460** | **-.450** | **-.473** | **-.316** | **-.360** | **-.453** | -.120 |
|  | *p* | .000 | .000 | .000 | .017 | .006 | .000 | .374 |
|  |  |  |  |  |  |  |  |  |
| **IMT** | *r* | **-.221** | **-.248** | -.165 | -.124 | -.158 | **-.388** | -.142 |
|  | *p* | .005 | .003 | .105 | .305 | .116 | .001 | .221 |
|  |  |  |  |  |  |  |  |  |
| **MPI** | *r* | **-.346** | **-.291** | -.121 | **-.298** | -.186 | **-.422** | -.042 |
|  | *p* | .003 | .013 | .234 | .010 | .116 | .000 | .723 |

**The analyses were adjusted for age and sex. IMT=Intima Media Thickness, MPI=Myocardial performance index**

**Supplementary table 2 .Simple correlation analyses between Adiponectin, TNF-α, MPI and NF-kB DNA methylation (n=356)**

|  | | **Mean NFKB Met** | **Met- pos 1** | **Met- pos 2** | **Met- pos 3** | **Met- pos 4** | **Met pos 5** | **Met- pos 6** | **Met- pos 7** |
| --- | --- | --- | --- | --- | --- | --- | --- | --- | --- |
| **Adiponectin** | *r* | **.325** | **.336** | **.235** | .080 | .094 | .096 | .094 | **.426** |
|  | *p* | **.003** | **.001** | **.001** | .165 | .103 | .098 | .104 | **.002** |
|  |  |  |  |  |  |  |  |  |  |
| **TNF alfa** | *r* | **-.273** | **-.183** | **-.279** | **-.179** | **-.173** | **-.167** | -.149 | **-.192** |
|  | *p* | **.006** | **.026** | **.001** | **.029** | **.036** | **.042** | .071 | **.049** |
|  |  |  |  |  |  |  |  |  |  |
| **IMT** | *r* | **-.238** | **-.265** | **-.288** | -.098 | -.104 | **-.325** | -.142 | **-.278** |
|  | *p* | **.004** | **.003** | **.005** | .178 | .195 | **.001** | .221 | .**003** |
|  |  |  |  |  |  |  |  |  |  |
| **MPI** | *r* | **-.303** | **-.358** | **-.271** | -.069 | -.186 | -.116 | -.042 | **-.328** |
|  | *p* | **.003** | **.001** | **.003** | .562 | .116 | .173 | .723 | .**000** |

**The analyses were adjusted for age and sex. IMT=Intima Media Thickness, MPI=Myocardial performance index**
